# Supplementary material for: Ameliorative Effect of Coenzyme Q10 on Phenotypic Transformation in Human Smooth Muscle Cells with FBN1 Knockdown
Source: Int J Mol Sci. 2024 Feb 25;25(5):2662. doi: 10.3390/ijms25052662 (PMC10931635; doi:10.3390/ijms25052662)
Supplement: Supplementary file 1 [file ijms-25-02662-s001.zip › ijms-2845853-supplementary.pdf]

Supplementary materials

Ameliorative effect of coenzyme Q10 on the phenotypic transformation in human smooth muscle cells with FBN1-knockdown

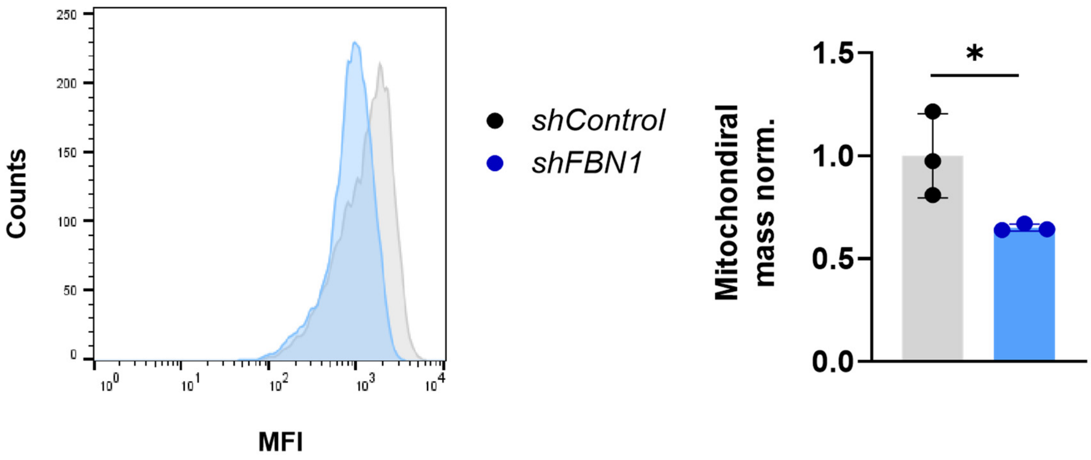

Figure S1. Mitochondrial mass (left) and corresponding statistical analysis (right) in *shControl* and *shFBN1* SMCs. **P* < 0.05. The *P* value was calculated by two-tailed Student's t-test. The numbers for the analysis were 3.

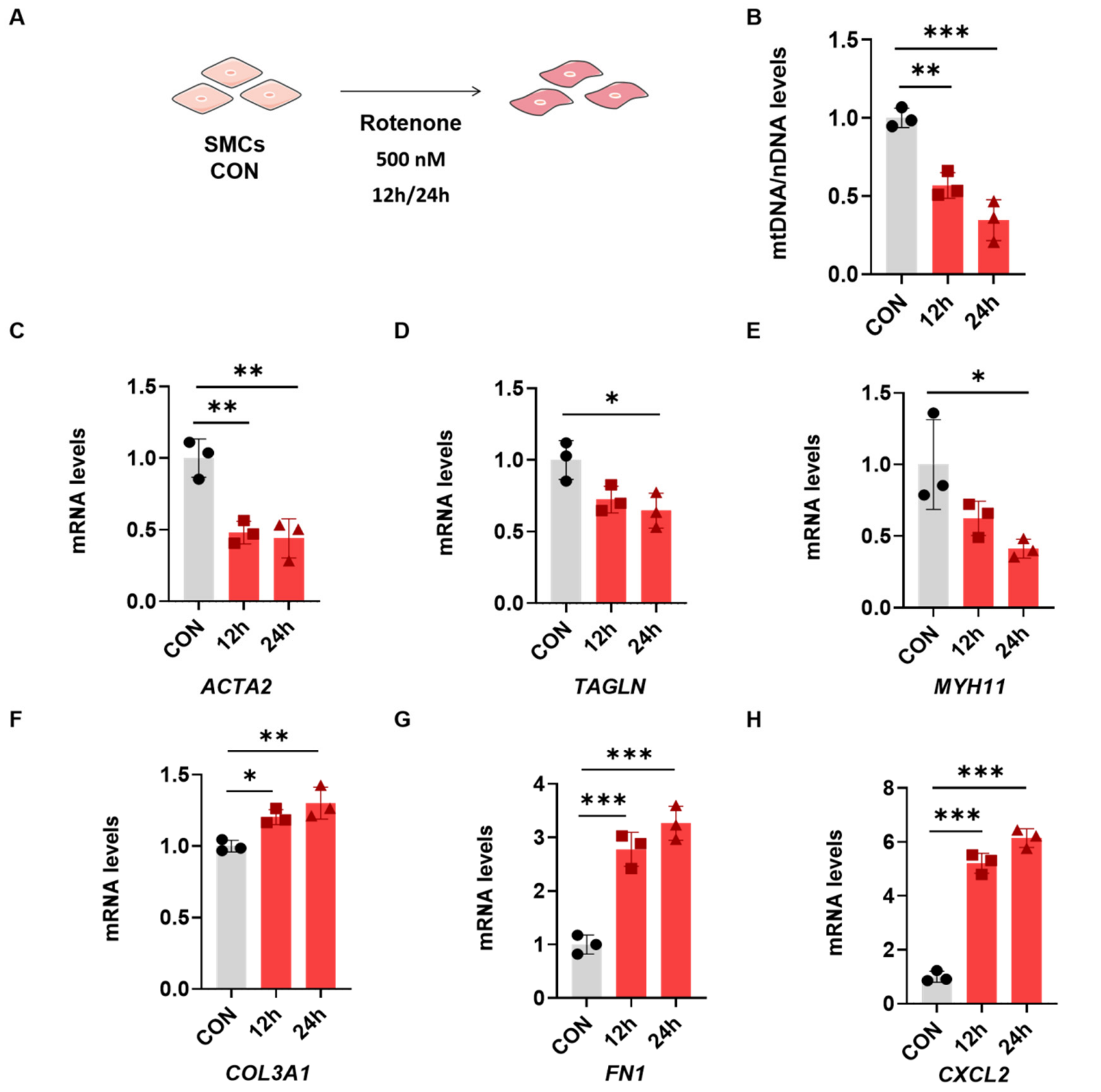

Figure S2. 500 nM rotenone-induced phenotypic switch of human smooth muscle cells. (A) Schematic of rotenone treatment. Quantitative reverse transcription PCR analysis of mtDNA content (B), *ACTA2* (C), *TAGLN* (D), *MYH11* (E), *COL3A1* (F), *FN1* (G) and *CXCL2* (H). *$P < 0.05$, **$P < 0.01$, ***$P < 0.001$. The *P* value was calculated by one-way ANOVA. The numbers for the analysis were 3.

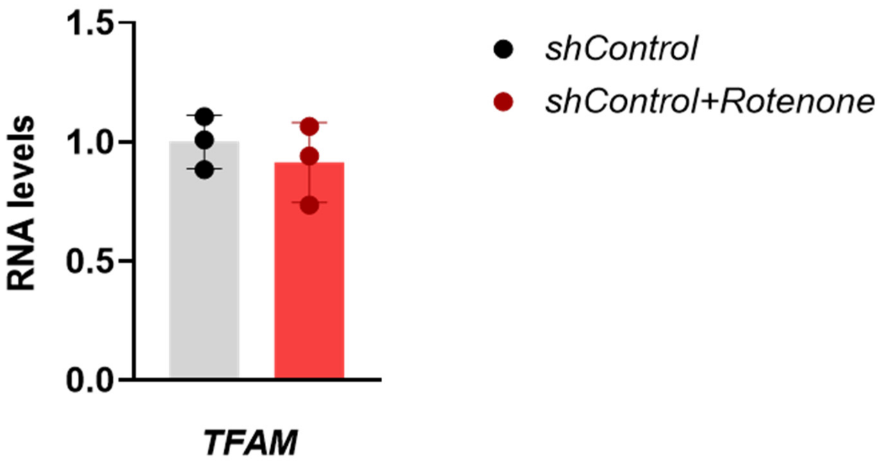

Figure S3. 1 μM Rotenone have no significant effect on *TFAM* expression levels in *shControl* SMCs after 24h treatment.

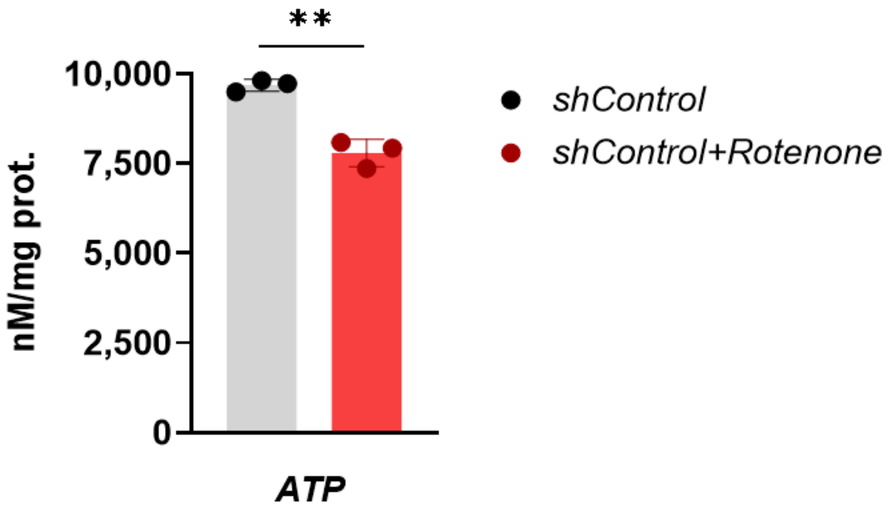

Figure S4. 1 μM Rotenone induced ATP level decline in *shControl* SMCs after 24h treatment.

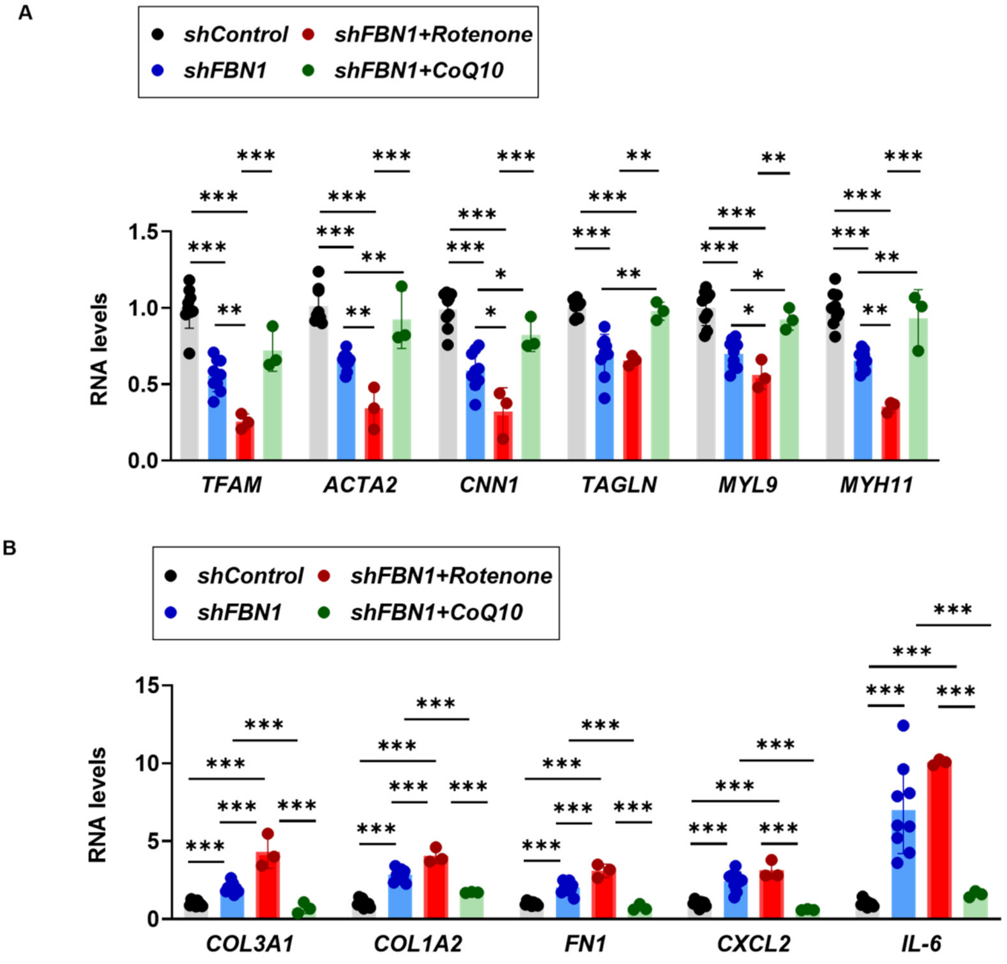

Figure S5. Summary of the transcriptional levels of *TFAM* (A), contractile (A) and synthetic (B) markers in SMCs with *shFBN1*, rotenone and CoQ10 treatment. *P < 0.05, ***P < 0.001. The P value was calculated by one-way ANOVA. N = 9 biological replicates for *shControl* and *shFBN1* groups, n = 3 biological replicates for *shFBN1*+rotenone and *shFBN1*+CoQ10 groups.

Table S1. Real-time quantitative PCR primers.

Gene	Forward (5’-3’)	Reverse (5’-3’)
**mRNA expression level**		
ACTA2	CTATGAGGGCTATGCCTTGCC	GCTCAGCAGTAGTAACGAAGGA
CNN1	GTCAACCCAAAATTGGCACCA	ACCTTGTTTCCTTTCGTCTTCG
COL1A2	GCAGGAGGTTTCGGCTAAGT	GCAACAAAGTCCGCGTATCC
COL3A1	GCCAAATATGTGTCTGTGACTCA	GGGCGAGTAGGAGCAGTTG
CXCL2	TTCACAGTGTGTGGTCAACAT	TCTCTGCTCTAACACAGAGGGA
FBN1	GCCGCATATCTCCTGACCTC	GTCGATACACGCGGAGATGT
FN1	ACAAGCATGTCTCTCTGCCA	TCAGGAAACTCCCAGGGTGA
GAPDH	CATGAGAAGTATGACAACAGCCT	AGTCCTTCCACGATACCAAAGT
IL-6	ACTCACCTCTTCAGAACGAATTG	CCATCTTTGGAAGGTTCAGGTTG
MYH11	CGCCAAGAGACTCGTCTGG	TCTTTCCCAACCGTGACCTTC
MYL9	CGAGGATGTGATTCGCAACG	TGTTTGAGGATGCGGGTGAA
TAGLN	CCGTGGAGATCCCAACTGG	CCATCTGAAGGCCAATGACAT
TFAM	AGAGCAGTCTGGGAGTAGGG	TGCTGCATTTGTCCCGAGAT
**mtDNA copy number**		
B2M	TGCTGTCTCCATGTTTGATGTATCT	TCTCTGCTCCCCACCTCTAAGT
mtDNA	CACCCAAGAACAGGGTTTGT	TGGCCATGGGTATGTTGTTA

Table S2. The *shFBN1* sequence.

**Gene**	**Vector**	**Sequence (5’-3’)**
FBN1-homo	Lentivirus	GCTGGTGGTGAGTGTATTAAC
